# Evaluating a Family Capacity-Building Service: Are We Doing More Good Than Harm?

**DOI:** 10.1177/00084174251323729

**Published:** 2025-03-13

**Authors:** Marie Grandisson, Myriam Chrétien-Vincent, Gabrielle Pratte, Cynthia Fauteux, Justine Marcotte, Emmanuelle Jasmin, Élise Milot, Julie Bergeron

**Keywords:** Capacity building, Family practice, Occupational therapy, Ergothérapie, renforcement des capacités, soins axés sur la famille

## Abstract

**Background.** Parents of children with special needs are more likely to experience stress and have health-related problems. Pediatric occupational therapy interventions that build parents’ capacity are often considered to be effective. It remains unclear how they can be offered without overburdening parents. **Purpose.** The purpose of this article is to share the findings from the evaluation of a flexible capacity-building occupational therapy service with seven families. **Method.** A convergent parallel mixed methods design was used to document parents’ and occupational therapists’ perspectives on the services, including outcomes, strengths, weaknesses, opportunities, and threats. **Findings.** Parents reported understanding their children better, having more positive attitudes toward the challenges experienced, feeling more confident that they could help them, and having more satisfactory family routines. The importance for therapists to develop nonjudgmental collaborative relationships, to be flexible and to use the time available to help families with what matters the most in their daily lives came out particularly loudly. **Conclusion.** This study provides a concrete example of how it is possible to build families’ capacities without overburdening them. It also provides guidance to establishments wishing to take a step back to think about how they build families’ capacities.

## Résumé

**Description.** Les parents dont les enfants ont des besoins de soutien particulier sont plus nombreux à expérimenter du stress et à percevoir leur santé négativement. Plusieurs interventions ergothérapiques destinées à renforcer les capacités des parents sont considérées efficaces. Or, il n’est pas évident comment elles peuvent être offertes sans surcharger les parents. **But.** Le but de cet article est de partager les résultats de l’évaluation d’un service d’ergothérapie flexible axé sur le renforcement des capacités des familles et ayant été offert à 7 familles. **Méthodologie.** Des méthodes mixes ont été utilisées à l’intérieur d’un devis convergent pour document les perspectives des parents et des ergothérapeutes sur les services, notamment sur leurs retombées, leurs forces, leurs faiblesses, les opportunités et les menaces. **Résultats.** Les parents ont rapporté mieux comprendre leur enfant, avoir une attitude plus positive face aux défis, se sentir plus confiants et avoir des routines familiales plus satisfaisantes. Les recommandations qui émergent font ressortir l’importance de développer des relations collaboratives dans lesquelles les parents ne se sentent pas jugés, d’offrir de la flexibilité et d’utiliser le temps disponible pour ce qui fera le plus de différence dans le quodien des familles. **Conclusion.** Cette étude offre un exemple concret concernant comment il est possible de renforcer les capacités des familles sans les surcharger. Elle offre aussi des pistes pour soutenir les établissement à prendre un pas de recul pour réfléchir aux services proposés aux familles.

## Introduction

Occupational therapists can use different types of intervention to build the capacity of significant adults involved in children's lives, such as parents and educators. These include (a) sharing general or individualized information with the adult; (b) doing activities with both the child and the adult, such as observation, modeling, or cointervention; and (c) focusing on problem-solving with the adult, such as coaching or expert consultation (Chrétien-Vincent et al., 2023a). Many of these interventions are documented to be effective in supporting children's development and participation and in increasing adults’ knowledge and sense of competence (Gagnon et al., 2022; Graham et al., 2013; Jeong et al., 2021; Novak & Honan, 2019). However, these interventions often require much commitment and time from adults.

Parents of children with special needs are more likely to experience frequent and intense stress, to have more health-related problems than parents of typically developing children, and to feel overwhelmed with daily routines (Beaudoin & Pratte, 2022; Miodrag et al., 2015). For example, mothers are significantly more at risk of being diagnosed with depression, anxiety, sleeping problems, and even musculoskeletal problems than mothers of children without disabilities (Brekke & Alecu, 2023). While rehabilitation professionals may provide services that are specifically oriented at providing psychological support to families (King et al., 2017), there has been insufficient attention in the literature so far on how not to overburden families with pediatric rehabilitation services that are aimed at building their capacity to help their children.

Family-centered practice provides some indications. Kokorelias et al. (2019) identified four key components of family-centered care: (a) collaboration between family members and healthcare providers; (b) consideration of family contexts; (c) dedicated policies and procedures; and (d) patient, family, and healthcare professional education. Phoenix et al. (2020) highlighted the importance of considering parents’ feelings, skills, knowledge, values, and beliefs. These authors also pointed out the importance of parents’ relationship with professionals and of being mindful of the logistics involved when asking families to attend rehabilitation sessions in the clinic. Solution-focused techniques may also be useful to build on families’ strengths and resources, and to promote a more positive outlook on a situation (Franklin et al., 2017; Neipp et al., 2021). Given that the main coaching approaches used in pediatric rehabilitation are aligned with these family-centered, collaborative, and solution-focused components (King et al., 2023; Schwellnus et al., 2015), the team expected that coaching would be part of the solution.

While this guidance is helpful, it can be particularly challenging not to overburden families when the services’ aims are explicitly to build their capacity to facilitate children's participation. To address this, a participatory action research project was conducted with a parent and a service coordinator from a not-for-profit social occupational therapy clinic whose mission was to provide accessible and affordable services to the population who most needed them in the Quebec City region (Québec, Canda). The team's goal was to codevelop, implement, and evaluate a capacity building occupational therapy service for families of children with special needs that does not overburden them. While occupational therapy services were offered at that clinic before the project, the goal was to develop a new capacity-building service for families that would eventually be accessible to all those in need. The team's desire was for the service to respond to the needs of families with diverse profiles (e.g., education, income, family structure, immigration status, place of residence, and ethnicity), including those more at risk of encountering barriers to services.

In the project's first phase, community forums were conducted with parents and occupational therapists (Grandisson et al., 2023). Key messages were identified regarding how professionals can build families’ capacities without overburdening them. These include facilitating quick and easy access to services; being sensitive to possible negative impacts of services; proposing flexible conditions; prioritizing objectives and interventions with the child and their family; avoiding giving too much information or too many recommendations; and making sure to highlight the positive elements, such as strengths, progress, or efforts. It also pointed out the necessity of taking sufficient time to develop a relationship, explain interventions, and conduct follow-ups. Furthermore, families and occupational therapists suggested that it is essential to collaborate not only with the family, but also with the different healthcare providers and to offer support in implementing the proposed activities or accessing the required equipment (Grandisson et al., 2023). The research team (including the parent–partner), along with an advisory committee composed of occupational therapists, a care coordinator, and an administrator from a social occupational therapy clinic, drew upon critical findings from the forums, as well as its own experiential, professional, and scientific knowledge, to develop an accessible, viable, and flexible capacity-building service for families of children with special needs. To do so, a group meeting was held online using the *Timeline exercise* (Chevalier et al., 2021) to identify key components of the service at different points (i.e., before, at the beginning, throughout, at the end). Additional individual contacts were made to validate different elements (e.g., to revise the proposed entry questionnaire).

### Description of the Capacity-Building Service Developed

The capacity-building service was called «FORCES» (which can be translated into strengths) because it focused on families’ strengths and resources. The service included a maximum of eight one-hour occupational therapy sessions offered between August 2022 and April 2023. It focused on building families’ capacities to facilitate their children's participation or development in a family-centered and solution-focused process. While no specific coaching approach was imposed, the occupational therapists were encouraged to pay attention to the relationship they developed with the family and to collaborate with them to identify the goal, to analyze the situation, to generate solutions, and to see if they worked. In other words, they were asked to use a coaching approach, to act as “collaborative facilitators who assist clients and families with their own discovery of solutions that fit their everyday contexts” (King et al., 2023, p. 1). To optimize the chances of building families’ capacities without overburdening them, the occupational therapists committed to being reflective and to trying to integrate the key principles identified in the previous phase of the study (Grandisson et al., 2023). After four sessions, parents were also asked to complete a similar feedback form.

Before the first session, families completed a short questionnaire to facilitate matching with an occupational therapist whose availabilities and expertise corresponded to their needs. The Canadian Occupational Performance Measure (COPM; Law et al., 2014) was used in the first and last sessions to identify priority areas and to document the children's performance and parents’ satisfaction in these priority areas. At the end of the first session, a menu of services (Appendix A) was used to facilitate discussions regarding objectives and modalities and to co-create the intervention plan. The menu included diverse options for Sessions 2 to 8, including sharing information on potential workshops, videos or readings, coaching with parents (virtual or at home), interventions with the parents and the child such as observing, modeling, cointervention or home programs, and further assessment of the child's abilities. The menu of services also included reminders to discuss the location of the service (e.g., at home, virtual, or at the clinic), its timing and frequency, partners to involve (e.g., school or daycare) and preferences for follow-ups (e.g., emails, phone). Parents were told that the child did not have to be present all the time and that one parent or both could attend.

To maximize time spent helping families with their daily challenges, other than the COPM (Law et al., 2014), no further assessment of the child's participation and abilities was conducted systematically at the beginning of the services. Yet, conducting further evaluations to better understand their child was included in the options of the menu of services. For the same reason, no evaluation report was provided upfront. After the last session, a more detailed note was sent to parents. It included a portrait of the child's participation in priority areas before and after the sessions, an analysis of factors facilitating and hindering participation in chosen occupations, and a list of supportive strategies.

The three occupational therapists who delivered the services had prior experience and/or training in coaching, including with Occupational Performance Coaching (Graham et al., 2013), Contextual intervention (Dunn et al., 2012) and Solution-focused coaching (Baldwin et al., 2013). Two of them had been involved in graduate studies using coaching approaches. One 2-hr virtual training was provided by team members before service delivery to help therapists integrate the key messages identified in the previous phase of the study (Grandisson et al., 2023), the COPM (Law et al., 2014) and the menu of services (Appendix A). A reflective exercise was proposed to therapists before and at mid-point during services to help them stay focused on the key elements identified to build families’ capacity without overburdening them (Appendix B). Templates for efficient note-taking were also provided. During the service, frequent virtual meetings were offered to support the occupational therapists and ensure the interventions aligned with the key principles. They were also provided with an inventory of informational resources that could be shared with parents as needed regarding common information needs.

### Research Questions and Objectives

The service was experimented with seven families in 2022–2023 to explore how it contributes to building the capacity of diverse families and whether it overburdens them. The research questions were: What are the resources needed to implement this service? What are the positive and negative outcomes of the service for children and families? What are the service's strengths, weaknesses, opportunities, and threats? This article's objective is to share the findings from its evaluation, including the resources required, the outcomes and the main lessons learned.

## Method

### Study Design

The overarching study is a participatory action research, which is suited to developing innovative solutions to problems for which scientific data are insufficient (Chevalier et al., 2021). A convergent parallel mixed methods design (Creswell & Clark, 2011) was chosen to evaluate the services developed in this project. Quantitative and qualitative data were collected to draw from their strengths and obtain a rich overall interpretation (Creswell & Clark, 2011). Ethical approval was obtained prior to the study from the Comité d’éthique de la recherche sectoriel en réadaptation et intégration sociale of the CIUSSS de la Capitale-Nationale (project #2021-2106, RIS).

#### Learning while doing

In line with the iterative and reflexive aspects of the participatory action research approach of the overall study (Bradbury, 2015), adjustments to the service were made during its implementation. To foster learning and alignment with principles to build families’ capacities without overwhelming them, after four sessions, parents and therapists completed a short form developed with findings from the previous phase of this project (Grandisson et al., 2023). Both forms included nine questions with a response scale from 1 to 4 (0: *not applicable*; 1: *not at all*; 2: *a little*; 3: *moderately*; 4: *a lot*). The forms completed by parents and therapists confirmed that the services aligned well with the critical messages identified to build families’ capacity without overwhelming them (see Appendix B). However, after four families completed four sessions, the feedback pointed out the need to support families further in implementing changes and to work more with school and daycare staff. The team clarified that if what helps a family is that the education setting receives assistance, therapists could use one or more sessions to go to daycares or schools and that a written summary of strategies to try should be provided as much as possible. Also, it was then deemed necessary to always schedule the following session at the end of the meeting to avoid wasting too much time trying to reach each other. Families were invited to cancel and reschedule if necessary.

### Participants

To be included in the study, families needed to be interested in services aimed at building their capacity to facilitate the participation of their child. Families were mainly recruited from the waiting lists of two partner organizations: (a) a social occupational therapy clinic and (b) an early intervention program from the public sector for children aged seven or under at risk of presenting developmental or participation challenges. The social occupational therapy clinic and the early intervention program typically serve children who do not have a specific diagnosis. Yet, whether their child was diagnosed or not was not an exclusion criterion. Families were contacted by representatives of partner organizations and introduced to the project by phone. When they accepted, a member of the research team called them to further explain the capacity-building service and the research project by phone. They were then sent the consent form by email if they were interested. One family already receiving traditional services at the social occupational therapy clinic was also invited. This family had initially been referred to services by the social pediatrics clinic of the region and was offered a new type of occupational therapy service. Families were told they could remain on the waiting lists or return to their original way of receiving services if they still had needs following their participation in the project. However, they could not receive two types of occupational therapy services for their child at the same time.

### Data Collection

Different tools were used to document parents’ and occupational therapists’ perceptions regarding the services. They are described below, starting by the quantitative measures, and following up with qualitative measures.

#### Canadian occupational performance measure

The French version of the COPM (Law et al., 2014) was completed with each family in the first and last sessions to measure the outcomes of the services on the child's participation. More precisely, the COPM uses an interview to identify intervention goals and a visual scale (1 to 10) to document changes in the child's occupational performance and parents’ satisfaction regarding the child's performance. The COPM has been adapted for studies with parents of children between 2 and 8 years of age and offers good construct and criterion validity and interrater agreement in this context (Cusick et al., 2007). It has been used in numerous studies with parents (e.g., Camden et al., 2021; Gagnon et al., 2022). As the service targeted parents’ capacity to promote their children's participation, the changes in the parents’ perceptions were considered sufficient to discuss the effects without specific child-focused measures.

#### Service statistics kept by the occupational therapists

To document the resources needed to implement the service, occupational therapists used an Excel document to record the distance between their homes and the location of the service, as well as the time spent on different types of tasks. It included time spent in preparation, meetings, traveling, note-taking, and follow-up phone calls or emails.

#### Measure of process of care

The measure of process of care (MPOC) was used at the end of the services to determine the extent to which the service was family-centered. More precisely, parents completed the MPOC-20 (King et al., 2004), while occupational therapists completed the MPOC for service providers (Woodside et al., 2001). French versions were used. Both are self-reported questionnaires using a seven-point scale, where seven represents the highest score (i.e., to a very large extent) and one represents the lowest score (i.e., not at all in the MPOC-SP, never in the MPOC-20). Both questionnaires have been validated and used in different contexts and were completed online using Limesurvey.

#### Parent's individual interviews

One parent per family participated in an individual semistructured interview virtually or by phone after the end of the services. The interviews were conducted by two team members (MCV and CF). They lasted between 9 and 34 min. These enabled the team to document their perceptions of the services’ outcomes (on their children, themselves, and their families), strengths, and weaknesses, as well as their suggestions for improvements. To evaluate whether the service was successful at building capacity without overburdening families, parents were also asked whether the service had had any negative consequences (unintended outcomes) for themselves, their child, or their family, and if so, to describe those consequences. Parents were also asked whether they felt that a larger initial assessment would have been beneficial.

#### Occupational therapists’ discussion group

At the end of the project, a discussion group was conducted with the three occupational therapists who implemented the services. It lasted 2 hr and was comoderated by a researcher (MG) and a citizen-partner (CF). Their perceptions were sought regarding the main strengths and weaknesses of the services, opportunities to make them accessible to a vast array of families while remaining efficient, and potential obstacles to reaching this goal.

### Data Analysis

Congruent with the convergent parallel design chosen (Creswell & Clark, 2011), quantitative and qualitative data were first analyzed separately and then combined to obtain an overall interpretation of outcomes and lessons learned. A weaving approach enabled the team to combine the insights from the qualitative analyses with those from the quantitative data analyses on a theme-by-theme basis (Fetters et al., 2013).

Descriptive quantitative analyses were done with the findings from the statistics kept by the occupational therapists and from the COPM (Law et al., 2014). In the COPM, the differences between the first and last sessions in the performance and satisfaction scales were measured for each priority area. A mean of these differences was calculated for each family. A change of two points in performance and satisfaction was considered clinically important, in line with previous studies with pediatric populations (Cusick et al., 2007). However, new perspectives published recently (McColl et al., 2023) have suggested that what is the minimal change considered to be clinically important might vary across populations and participants. In this study, a combination of the COPM results with qualitative data regarding parents’ perspectives of the outcomes of the services was used. As for the MPOC (Woodside et al., 2001), the means for each subtest were considered for analysis.

The interviews and discussion group were carried out in French, recorded, and transcribed. A content-analysis approach was used. Data first was coded deductively to determine if the segments were about the outcomes of the service, its strengths, its weaknesses, or about opportunities or threats (SWOT). Then, an inductive approach was used to discover key themes in the data for each of these categories. The key themes identified in the SWOT were then merged into lessons learned on how to build diverse families’ capacities without overwhelming them by identifying relationships between the different themes. Following Patton's (2015) recommendations for analytical triangulation, two members of the research team (MCV and MG) read the transcripts from three interviews to identify key themes. They met to compare them, highlighting similarities and differences, and to develop the coding tree together, along with preliminary definitions. One of them coded the interviews into NVivo software. The other performed a progressive of all the content. The key insights were then presented to the team for further validation and to clarify which key lessons learned should be emphasized. At this point, the team considered whether some of the quantitative data collected (i.e., MPOC, service statistics and form completed at midpoint) also supported the key lessons, or whether it contrasted with them. The chosen excerpts were translated into English by a professional.

## Results

### Participants

Eight families provided informed consent to participate in the study and one withdrew prior to the first meeting with the occupational therapist because the parent perceived that their child was doing well enough. When asked whether they identified as belonging to an underrepresented minority, only one (F6) of the seven families said yes (for immigration and skin color). One family lived in a rural area (F5), while all the others resided in Quebec City (Canada). The main characteristics of the children of the included families are presented in Table 1. Their age varied from 3 to 8 years of age. Initial reasons for being on a waiting list for services varied widely.

**Table 1.** Characteristics of the Children.

	Gender	Age at the beginning of the service (years; months)	Initial reasons for occupational therapy consultation
F1	M	8 y; 3 m	Difficulties initiating activities (going to school, bathing, getting ready for bed).
F2	F	8 y; 10 m	Hypothesis of hyperreactivity (clothing label) and sensory overload leading to outbursts.
F3	F	6 y; 11 m	Difficult sleep, insufficient strategies to self-regulate. More hyperreactivity when anxious.
F4	M	5 y; 9 m	Concerns related to school entry (structure and sensory stimulations). Dressing and morning routine.
F5	M	3 y; 6 m	Adaptation challenges, rigidity, repetition, intense tantrums, tics.
F6	F	4 y; 10 m	Food selectivity and reactions to food textures.
F7	F	5 y; 3 m	Fine and gross motor skills, particularly preschool skills.

### Services Delivered

The services were offered over a period ranging from 106 days to 211 days (*M* = 151 days or about 5 months). A portrait of the services offered and the proportion of direct time invested is presented in Table 2.

**Table 2.** Portrait of the Services Offered and Proportion of Direct Time.

Family	Number of meetings	Location of meetings			Total direct time (hr: min)	Total indirect time (hr: min)	% direct time
		Virtual	Home	Educational setting			
F1	8	1	7	0	8:15	10:05	45
F2	5	5	0	0	5:40	4:55	54
F3	8	3	4	1	12:35	9:10	58
F4	8	4	1	3	12:30	9:35	57
F5	8	8	0	0	10:05	4:50	68
F6	5	5	0	0	4:30	3:05	59
F7	5	1	2	2	6:05	5:50	51
*M*	6.7	3.9	2	0.9	8:31	6:47	56

A total of 47 sessions were delivered for a mean of 6.71 sessions per family. Over half of them were virtual or phone coaching (*n* = 26/47). Coaching, observation, and modeling in the activity with the child and a significant adult were done at home (*n* = 15/47) or in education settings (*n* = 6/47). Three families only used virtual coaching, two of them by choice (F2 and F6) and one because they lived in a remote region, far from the therapist (F5). The child was directly involved in fewer than half of the meetings (*n* = 19/47). Only one standardized assessment of a child's abilities was carried out in response to a parent's concerns (F3). The rhythm of the services varied greatly: some met every 2 weeks; others decided to start more intensively and gradually leave more time between sessions. A mean of approximately 15 hr of occupational therapy was invested for each family. Some families had many direct contacts with therapists outside the sessions (i.e., phone, emails). A more significant proportion of direct time was found in families with eight meetings and those who only did virtual meetings. The occupational therapists spent a mean of 4 hr of note-taking for each family.

Parents’ and occupational therapists’ perspectives regarding the extent to which the services respected the key messages regarding how to build families’ without overburdening them (Grandisson et al., 2023) are available in Appendix B. Similarly, their perspective regarding the services in the MPOC (Woodside et al., 2001) are presented as in Appendices C and D. While therapists tended to evaluate their services more critically, parents were generally very positive suggesting high fidelity of the service with key messages on how to build families capacities without overburdening them and key elements of family-centered practice.

### Outcomes of the Services

In the interviews, all parents reported experiencing positive personal changes and changes in their children following their involvement in the services.

#### It generally did not overwhelm families

When questioned whether the services negatively affected their families, all but one answered that they had not. While being very satisfied with the services, that parent affirmed that at a certain point, adding sessions to their busy schedule was demanding for parents, so they decided to take a longer pause over the holidays. In the feedback forms completed at mid-point, all parents indicated that therapists were “a lot” sensitive to the potential negative impacts of the services for the families. Three parents explained how they perceived the services as less stressful or more fun than traditional ones for them and their children. One expressed how their child was motivated to progress as he knew the therapist was coming and shared how “he was always happy to show her his calendar and the efforts he'd made on his own” (F1). Another explained how, their child did not even know they were involved in occupational therapy:

In the end, my daughter didn't join in, so she doesn't even know that I've had occupational therapy sessions …; for her, it's mum playing games with her, then doing activities, and everything is just fine, like that, I think. It's less stressful, maybe for her too. (F6)

#### It boosted parents’ confidence in their capacity to help their children

All parents mentioned feeling more confident about helping their children following their engagement in the services. For example, one said, “It gave me a boost … it gave me confidence … it didn't change the boy he is, but it changed the way we deal with him” (F5). They explained that the services helped them to understand their child's needs and reactions better and to identify strategies that help them. They could then better support their child's participation at different moments of the family routine. One parent affirmed that “I can assist her better, I'm more aware, I was less aware of how to act with her before … I know more what to do to encourage her to draw” (F6). One parent added that the strategies they identified with the occupational therapist boosted their confidence to address challenges in other situations (F1). Parents highlighted the fact that the occupational therapist encountered staff from educational settings was meaningful for them. One explained that “It gave us a kind of support. … We weren't just parents asking for things for our child; we were parents who were *right* to ask for things for our child” (F4).

#### It led to improved child participation and a more satisfying family routine

In the COPM (Law et al., 2014), a two-point improvement or more was found in at least one priority area for all children in both occupational performance and parents’ satisfaction scales. Considering all the priority areas identified, the mean differences between the first and last meetings ranged from 0.25 for one child to 6 for another (*M* = 2.4). The mean changes in performance were higher than two for four children (F1, F5, F6, and F7). Mean changes in parents’ satisfaction regarding their child's performance ranged from 1.4 to 6 (*M* = 3.1) and were higher than two for five parents (F1, F2, F3, F6, and F7). In the interviews, parents all expressed that the services led their child to participate in a more satisfying way in chosen occupations. They shared examples of how services led to improvements regarding how their child gets dressed (F4 and F5), eats a greater variety of food and/or with less mess (F2 and F6), takes their bath more frequently (F1), gets to sleep on their own (F1); engages in activities associated with drawing and cutting (F7); and has more positive relationships with siblings (F4). Many parents highlighted how this led to a more satisfying family routine for themselves and their children. One parent expressed feeling the services have “changed our (their) life” with their child (F5). They explained that the increased autonomy of their child and the fact that they felt they knew how to provide better support led to a greater enjoyment of family time for all concerned. Parents shared many examples of how their routine became more manageable and less stressful around meals, baths, and bedtimes, as well as during dressing. For example, one said:

She really eats like the rest of us, so it's made things a lot easier … it's less stressful for her, I think. … I cook up fewer little things for her on the side. (F6)

One family shared how they were finally able to enjoy their family holidays:

It's been years since we've had a peaceful holiday! There's always an escalation in tension …; it was so difficult during holidays, we cried bitter tears over vacations for years, and then this year, we kind of maintained the program at all costs; we maintained the meal times … she needs to stick with her routine, come what may; even if accommodating her takes energy, we also save a lot of hassle in the end. (F3)

#### It modified families’ attitudes toward the child

In five out of seven families, parents highlighted that the services helped family members modify their attitudes concerning the challenges experienced by the child. One parent said, “We feel very good with our little guy. … We didn't say that before” (F5). Two others expressed how the support received helped them be more patient and calmer (F4 and F6). Another shared that they realized how their child could be involved in implementing strategies that help them (F2). One parent explained how the therapist gradually helped them accept strategies typically used with autistic children:

Social scenarios … are tools I've already used with ASD (autistic) children, so it certainly strikes a chord with me when I'm asked to use these techniques with my own child …. (name of the therapist) helped me to accept using certain tools that are more often used with autistic people. … For me, it's been a … personal learning process to be able to move forward in this area and to understand that … this helps to relieve my daughter's anxiety. (F3)

One parent even shared how it fostered a more positive attitude in siblings:

My oldest always used to add her two cents worth, her little digs, you know, like … ‘(Child's name) is a bit weird, eh?’ … But then, the other day, she said to me, ‘Mom, (child's name) has really improved, hasn't he?’ I said, ‘Yeah, really! You got that right!’ (F5)

### Lessons Learned to Build Families Capacities Without Overburdening Them

The analysis of the resources needed to offer the service, as well as the perspectives of families and occupational therapists regarding the strengths, weaknesses, suggestions for improvement, opportunities, and threats of the service, enabled the team to identify seven key insights, or lessons learned to build diverse families’ capacities without overwhelming them.

#### Lesson 1. Develop a nonjudgmental and collaborative relationship with families

All parents highlighted that a very important strength of the service was associated with their relationship with the occupational therapist, especially how they felt comfortable with her and how they did not feel judged or pressured. For example, one shared:

We didn't feel any judgment about the fact that we hadn't really applied 100% of what we'd discussed the time before. It was really like a helping relationship with very few constraints … it was just positive precisely because of this very human connection. (F4)

Two parents explained how they liked being considered partners. The first said, “It was a team effort if you like, between what the occupational therapist can contribute and what we as parents know about the child” (F4). The second weighed in with this explanation:

It was never a situation where I saw someone prescribing something to us … the aim was to put us in a position of trust and then to equip us as parents … to talk as equals rather than someone coming in and saying 'Well, here, do this, do that, do this other thing.' (F2)

#### Lesson 2. Empower families to make informed decisions regarding services

Parents’ and therapists’ feedback suggests that it is essential to empower families to participate in decisions regarding which interventions are offered, and where, when, and how often. Parents appreciated that diverse options were offered, including virtual services and services at home, daycare, or school. Two parents explained how having virtual coaching sessions at times that suited them was helpful (F5 and F6). One of them said:

It was really helpful there, because with four children it would have been difficult to free up the two of us in the evening. We did it after the evening routine. The children were in bed. (F5)

All perceived the flexibility of the service as a key strength, something critical to families’ engagement. Therapists highlighted that a rigid organizational structure in which all families must receive the same service would be a significant barrier to implementing services that build capacity without further exhausting families. Results from the feedback form indicated that all parents and therapists felt the objectives and interventions were prioritized “a lot” with the child and his or her family. One parent and all the occupational therapists, however, pointed to the need to describe further what services can be offered to empower parents to make sound decisions. Occupational therapists suggested explaining each option's potential benefits and limits. Occupational therapists also emphasized the importance of not pressuring therapists to provide services at times that do not align with their realities. They suggested matching families with therapists whose availabilities are similar and favoring virtual services in the evening.

Therapists also highlighted that it should be clear that families can decide not to start the services, to wait a few weeks or months if needed. Two families (F2 and F5) and all the therapists expressed how offering services at the right intensity for a family can help facilitate their engagement. One therapist shared that while she favored letting families lead decisions regarding the rhythm of service and its adjustment, her experience in the project led her to recommend minimally setting the next appointment while remaining open to changing it if needed. She explained that when she did not schedule meetings ahead of time, she often ended up feeling like “the annoying occupational therapist who's always on their case.”

#### Lesson 3. Use the time available to make a significant difference for families

Quantitative and qualitative data indicate that the fact that the services were focused on finding and implementing solutions to the priorities in families’ daily lives was a critical strength. Both parents and occupational therapists highlighted that a major strength of the service developed was its efficiency in addressing families’ priorities. One therapist expressed how “the time goes directly into helping families …. The evaluation is effective, and so is the writing.” A parent mentioned:

I think it's the service we received for the family that was the most effective in a short space of time …. We discussed the issues right away, and what was really bothering us … became concrete right away. (F5)

Parents and therapists emphasized how they quickly determined the priority areas with families and focused on finding and implementing simple solutions to improve the child's participation. One therapist described the situation this way:

I think that families don't have much time, and we don't want to exhaust them … [T]he time they invest in putting things in place to make things better makes more sense than spending a lot of time being evaluated.

One therapist explained how doing home coaching facilitated the implementation of changes in the family's daily routine.

We'd go into the bathroom to check up on things and we’d say … ‘Okay, where do we put her positive reinforcement poster? Do we put it just over here at the side of the tub?’ … Then I'd head off and the strategies were all in place.

Another occupational therapist explained how going to the daycare and then to the home helped, as she and the child were able to demonstrate simple ways to foster engagement in graphic activities at home. She said:

The little girl and I said we'd show Mom what kind of stuff you do at daycare … It was really through the laundry pile. It was very informal. I'd say: 'Do you have some paper and pencils? … It's not a big song and dance; you don't have to get out a mega-patent for arts and crafts …' The mom was like 'Okay, okay, it's not complicated' … After that she said 'Okay but [now] she's always drawing. I've put up sheets for her'…

When asked whether a more comprehensive assessment would have been beneficial, almost all parents said no (*n* = 6/7). One parent explained her reasoning: “Well, you see, I don't have much time in my life, so taking time when I don't have any is a tough nut to crack” (F7). The only parent who said they would include a broader evaluation of the child's development claimed that this would allow her to see why they still needed occupational therapy as they were on a waitlist for services in the public sector due to a suspicion of developmental challenge (F3). Occupational therapists explained that in some cases, more comprehensive assessments are required, but not always. Quantitative data collected in the service statistics completed by the occupational therapists support the idea that the service was efficient, with a high proportion of therapists’ time spent in direct contact with the family (Table 2).

#### Lesson 4. Ensure support remains available for an extended period

All parents and therapists generally appreciated having the possibility of eight sessions over a period of approximately 6 months. However, many perceived the fact that the service had an end as a weakness. All three occupational therapists indicated feeling somewhat uncomfortable and recommended to at least “leave the door open a little longer.” Doing so could enable therapists to be available to talk on the phone, respond to questions by email, or even start a new session of services if needed. Parents and therapists mentioned this would be particularly useful if new needs emerge or the situation regresses. Therapists perceived that this would help parents feel more secure. One occupational therapist made the following point:

The support services ended abruptly. … I think that in an ideal world, there would be a session to provide them with tools. Then, after that, there might be a bit of a buffer period … So they don't feel, 'Well, I had this person there to reassure me and give me confidence, and then, bang! It's all over!’ If we could have just remained available without necessarily intervening, well, of course, the family would have felt a little more confident knowing that we were there and that if they had any questions, they could ask us again.

#### Lesson 5. Facilitate access to general information

Therapists suggest that it would be helpful to further facilitate the provision of general information to parents and other key actors in children's lives. While this is congruent with therapists’ lower evaluation of items in this category in the MPOC-SP (*M* = 1.27/7), no parent mentioned that when asked what could be improved. Yet, findings from the MPOC-20 indicate that parents felt therapists provided general information to a fairly great extent (*M* = 5.44/7). While the inventory of resources offered was perceived as relevant by therapists, it did not provide information to address all their needs, and they mentioned not being sufficiently familiar with it. They reported feeling limited by the fact that a great deal of information available is specific to a particular diagnosis, so it might be negatively perceived by many parents whose children have not received that diagnosis. An occupational therapist shared that it would probably have been more efficient to start by sharing key principles regarding mealtimes with the parent before doing coaching sessions to find strategies. Another stressed the importance for health services to provide such general information not only to parents but also to other significant adults in children's lives, such as educators from early childhood centers as well as school personnel.

#### Lesson 6. Favor interdisciplinary community-based services

In the discussion group, therapists perceived as an opportunity the provision of services to children, families, and educational settings within a particular geographic area close to the therapist's home base. They highlighted how this would help save time, travel, and costs. To illustrate these advantages, one therapist argued: “Of course, if you're close by, you travel less … the wheel keeps on turning.” They also stressed that they believed it would facilitate the development of relationships with families and community partners and enable the provision of the full range of services to all families, including virtual support and support in the different contexts in which the child evolves (e.g., home, daycare, school). Therapists noted that this would help offer more accessible services to a broader range of families. One therapist explained that some families might not ask for help, fill out different forms and procedures, or come to a clinic, that services should be provided in the community.

Occupational therapists suggested developing partnerships with different organizations involved in children's and families’ lives, such as daycares, schools, and community-based organizations. One therapist asked: “Do we really just want to build the capacity of families?” In cases in which therapists went to the daycare or schools upon requests from the families, the therapists explained how it was challenging and even uncomfortable sometimes as they came for one or two meetings only, focused on an individual child, and did not know the educators and teachers beforehand. Even though parents mentioned that they appreciated that support in the educational settings, therapists perceived the way it was offered as a weakness and wished for more teamwork and collaboration. One therapist explained:

To go to daycare but … to have to stay focused on the child that I'm going to see … Can I go to the other group too? Then, the other group? Can I meet all the educators? That's why they told me, 'Hey, you'll have to talk to everyone (at the daycare) about it' … It showed me the need to go beyond individualized services … it was the service centered on the family that held me back me a little.

Occupational therapists also stressed the need to partner with other professionals to offer services that build families’ capacities without adding to their load. They highlighted how exchanging with the psychoeducator was helpful for one family and how they would have liked to have had easier access to an interdisciplinary team.

#### Lesson 7. Support therapists and services administrators

To help bring change in practice, the three occupational therapists who delivered the services suggested providing training using findings from this study, demonstrating why this type of practice can be beneficial and providing ongoing support for therapists to help them gradually make changes in their practice. One therapist suggested presenting it as an inspiring example, rather than something that is easy or that should be implemented strictly. They also described how the fact that they could support each other or go to the principal investigator when they had questions helped them feel more confident in implementing the services. In addition, they suggested working with the administrators of organizations in which therapists offer services to point out the benefits of such practices, to make sure they offer the flexibility needed, and to help them document quality indicators regarding how the services made the families feel or what it brought to them. Regarding these indicators, one therapist said, “I'm (not) sure we can show that we'll see more of them, faster necessarily, but we'll serve them better.”

## Discussion

This study enabled the development of a capacity-building occupational therapy service that started by identifying families’ priorities, used a coaching approach and a menu of services to determine interventions and modalities, and integrated reflective practice and feedback from families. The evaluation of the service offered to seven families indicates that the service helped build families’ capacity without overwhelming them, suggesting the key messages (Grandisson et al., 2023) used to develop the service and to help therapists be reflective about the services were relevant. Children's participation increased in at least one priority area for all children, and parents shared many examples of how this led to more satisfactory family routines. Parents reported understanding their children better, having more positive attitudes toward the challenges experienced, and feeling more confident that they could help them. These findings suggest that the services contributed to fostering families’ resilience, changing their belief systems toward a more positive outlook, helping them accept what can't be changed and become more confident they can overcome challenges (Walsh, 2016).

Parents in the current study highlighted how crucial the collaborative and nonjudgmental relationship developed with the occupational therapist was for them. Therapists also suggested that it is important to make parents feel it is acceptable to take one step at a time and implement only what they can. This brings back the importance of being sensitive to the potential negative consequences of services such as making the family feel guilty of not doing enough (Grandisson et al., 2023). This also echoes Pozniak et al.’s (2024a) conclusions about parents wanting individualized services and to be treated with care and empathy. The current study provides a concrete example of how it is feasible to apply principles for family-centered practice without needing too much resources. The data collected on the resources needed are encouraging given the average direct time spent being 56%, the mean time of note-taking per family being 4 hr, and the positive outcomes documented for parents and children. Families particularly appreciated how quickly the occupational therapist could identify their everyday challenges and help them find solutions. Professionals should be mindful of the resources being mobilized, for the family and the healthcare system, when systematically doing initial large-scale assessments at the beginning of services. Nonetheless, as others have suggested, it requires a shift in how therapists see their role, from expert to collaborator (King et al., 2019; Schwellnus et al., 2020), or from evaluator to change maker.

Parents in the current study generally did not feel overwhelmed with the service, even though the service's aim was to build their capacity. This might be due to the attention paid to increasing therapists’ awareness that they might do harm with the services, to the nonjudgmental relationships developed and to the service flexibility. A recent study investigated the experience of healthcare providers who are parents of children receiving care (Pozniak et al., 2024b). It highlighted how having that lived experience had changed the way their practice, and especially the way they interact with families with more empathy, how they made sure not to add to their load, and how some advocated for the provision of services in the evenings and weekends. While therapists involved in the current study did not have that lived experience, the training, support, menu of services and the reflective exercise proposed might have helped them make sure they did more good than harm.

Parents involved in the current study particularly appreciated the service's flexibility, made possible through the menu of services. This reinforces the importance of avoiding one-size-fits-all solutions, validating that flexibility is key to providing capacity-building services that do not overburden families (Grandisson et al., 2023). While Pozniak et al. (2024a) indicate that parents want to be involved in determining the care plan, this study has demonstrated how they can be in the driver's seat regarding the types of services into which they are ready to invest time and energy and the conditions of the services. Our findings suggest that the collaborative approach used to set goals and service modalities was helpful. However, they point out the need for families to fully understand the available options for services and the pros and cons of each option to make informed decisions. For this to happen, Pellecchia et al. (2023) suggest that therapists using a coaching approach should be offered targeted training on collaborating with caregivers to make decisions, how to use their ideas, and how to set goals with them. While more and more evidence on the effectiveness of coaching families and educators involved with children, with or without diagnoses, is becoming available (Chrétien-Vincent et al., 2023b; Gagnon et al., 2022; Graham et al., 2013; Novak & Honan, 2019), this study reminds professionals of the importance of providing the right service for the right family at the right time. This might sometimes involve postponing the beginning of services, taking a break, or going beyond coaching sessions with parents to include activities done directly with the child or with actors from the educational setting.

The ability to provide services without overwhelming families not only relies on having trained and sensitive professionals; it also requires organizational policies to be supportive and services to be tailored to needs, not diagnoses (Pozniak et al., 2024). This study highlights the importance for professionals to take the necessary time to develop a trusting relationship with families and educators, to offer both in-person and virtual care, and to go to daycares or schools as needed. Building families’ capacity necessitates going beyond the family unit to see who else is present and significant in the children's lives (Poissant, 2014). In our study, occupational therapists felt that their actions in daycares and schools were limited because they were only there for one or two sessions and to help with a particular child. They did not have time to build trusting relationships with educators or to obtain a thorough understanding of the educational context in which the child evolved, elements which are essential for effective collaboration (Weglarz-Ward et al., 2020). Recommendations from a Delphi study highlight the necessity of providing population-based services in educational settings and of developing meaningful indicators of successful services that go beyond how many children are seen are needed (Pratte, 2024). These certainly require work and advocacy at the organizational level.

### Study Limitations

Nonetheless, the service was tested with only seven families, and those were not as heterogeneous as hoped. The research team relied on the partnership with the social occupational therapy clinic to identify families with diverse cultural backgrounds and socioeconomic status. However, the permanent clinic closing during the study led the team to search for other options in a very limited period. Future research could further explore whether and how such flexible capacity-building service might facilitate the engagement of families who tend to encounter more barriers to care. This is particularly critical as families from disadvantaged socioeconomic status, single parents, and people from culturally or linguistically diverse backgrounds typically encounter more barriers and receive less support (Boulton et al., 2023) and are more likely to have their children identified as having a vulnerability or delay (Ducharme et al., 2023).

## Conclusion

The occupational therapy service developed appears successful at building families’ capacities without overburdening them, at doing more good than harm for families. Its focus on action over assessment, its flexibility, and the nonjudgmental relationships developed with families, emerged as particularly critical. While this study has provided a concrete example of how it is possible to build families’ capacities without overburdening them, it is hoped that it will be considered as an inspiration, not as a recipe. Health professionals and administrators from a variety of care contexts are invited to take a step back to think about how they build families’ capacities, reflecting on whether they are asking too much from families, on whether they are using the time and resources available to make a significant difference for families, and on whether their service is accessible and meaningful to those who most need it.

## Key Messages

- The capacity-building service developed helped families become more confident they can help their children participate in a more satisfactory way in family routines.
- This study has provided a concrete example of how it is possible to avoid one-size-fits-all solutions in pediatric rehabilitation services while remaining sensitive to the available resources.
- The service's flexibility and its focus on quickly addressing families’ priorities were particularly appreciated by therapists and families.

## Supplemental Material

sj-docx-1-cjo-10.1177_00084174251323729 - Supplemental material for Evaluating a Family Capacity-Building Service: Are We Doing More Good Than Harm? Supplemental material, sj-docx-1-cjo-10.1177_00084174251323729 for Evaluating a Family Capacity-Building Service: Are We Doing More Good Than Harm? by Marie Grandisson, Myriam Chrétien-Vincent, Gabrielle Pratte, Cynthia Fauteux, Justine Marcotte, Emmanuelle Jasmin, Élise Milot and Julie Bergeron in Canadian Journal of Occupational Therapy

sj-docx-2-cjo-10.1177_00084174251323729 - Supplemental material for Evaluating a Family Capacity-Building Service: Are We Doing More Good Than Harm? Supplemental material, sj-docx-2-cjo-10.1177_00084174251323729 for Evaluating a Family Capacity-Building Service: Are We Doing More Good Than Harm? by Marie Grandisson, Myriam Chrétien-Vincent, Gabrielle Pratte, Cynthia Fauteux, Justine Marcotte, Emmanuelle Jasmin, Élise Milot and Julie Bergeron in Canadian Journal of Occupational Therapy

sj-docx-3-cjo-10.1177_00084174251323729 - Supplemental material for Evaluating a Family Capacity-Building Service: Are We Doing More Good Than Harm? Supplemental material, sj-docx-3-cjo-10.1177_00084174251323729 for Evaluating a Family Capacity-Building Service: Are We Doing More Good Than Harm? by Marie Grandisson, Myriam Chrétien-Vincent, Gabrielle Pratte, Cynthia Fauteux, Justine Marcotte, Emmanuelle Jasmin, Élise Milot and Julie Bergeron in Canadian Journal of Occupational Therapy

sj-docx-4-cjo-10.1177_00084174251323729 - Supplemental material for Evaluating a Family Capacity-Building Service: Are We Doing More Good Than Harm? Supplemental material, sj-docx-4-cjo-10.1177_00084174251323729 for Evaluating a Family Capacity-Building Service: Are We Doing More Good Than Harm? by Marie Grandisson, Myriam Chrétien-Vincent, Gabrielle Pratte, Cynthia Fauteux, Justine Marcotte, Emmanuelle Jasmin, Élise Milot and Julie Bergeron in Canadian Journal of Occupational Therapy
