# Supplementary figures and images for: Evaluating a Family Capacity-Building Service: Are We Doing More Good Than Harm?
Source: Can J Occup Ther. 2025 Mar 13;92(2):113–25. doi: 10.1177/00084174251323729 (PMC12117127; doi:10.1177/00084174251323729)

**Appendix A. Menu used to discuss with parents at the first session**


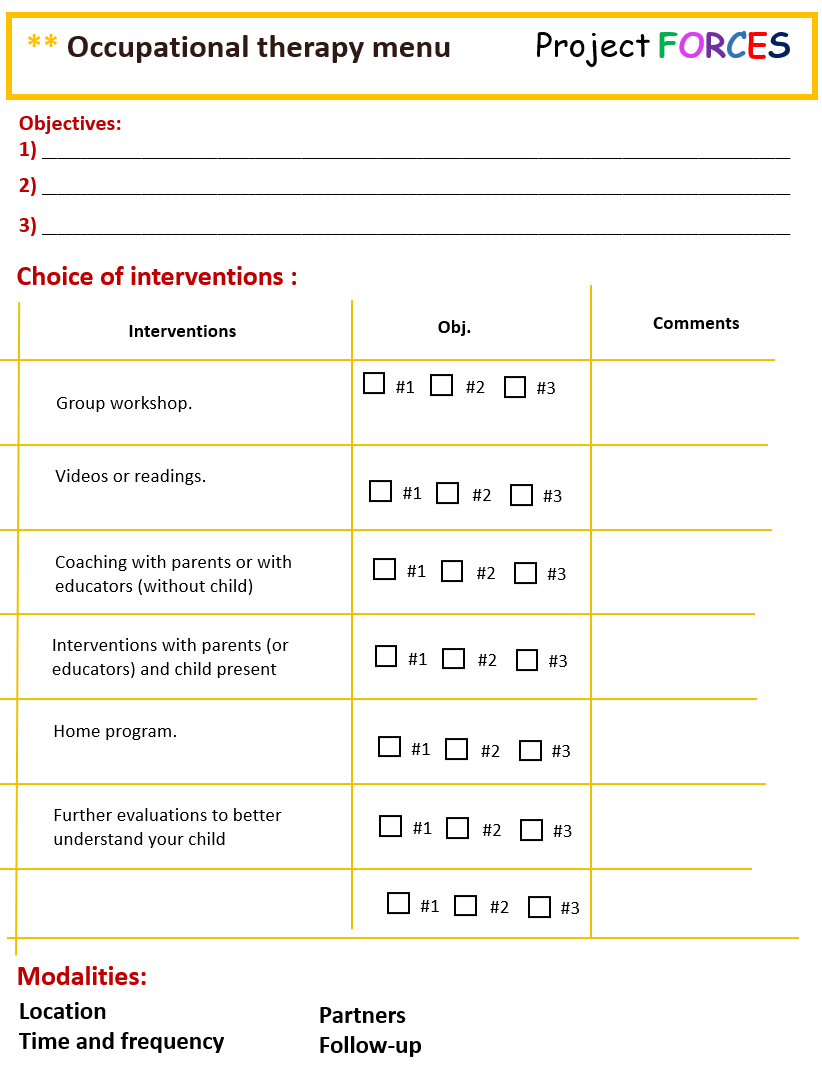

Supplement: sj-docx-1-cjo-10.1177_00084174251323729 - Supplemental material for Evaluating a Family Capacity-Building Service: Are We Doing More Good Than Harm? [file sj-docx-1-cjo-10.1177_00084174251323729.docx]
