# Supplementary material for: Evaluating a Family Capacity-Building Service: Are We Doing More Good Than Harm?
Source: Can J Occup Ther. 2025 Mar 13;92(2):113–25. doi: 10.1177/00084174251323729 (PMC12117127; doi:10.1177/00084174251323729)
Supplement: sj-docx-2-cjo-10.1177_00084174251323729 - Supplemental material for Evaluating a Family Capacity-Building Service: Are We Doing More Good Than Harm? [file sj-docx-2-cjo-10.1177_00084174251323729.docx]

**Appendix B.**

**Reflective practice and feedback forms completed by therapists and families after four sessions together**

| **To what extent do you feel that...** | **1**  **Not at all** | **2**  **A little** | **3**  **Moderately** | **4**  **A lot** | **I don’t know** |
| --- | --- | --- | --- | --- | --- |
| 1. Quick and easy access to services was provided. |  | 2 therapists | 1 parent  1 therapist | 6 parents  4 therapists |  |
| 2. Sensitivity was shown as regards the possible negative impacts of services on the child and his/her family. |  |  | 3 therapists | 7 parents  4 therapists |  |
| 3. Flexible arrangements were on offer. |  |  | 1 therapist | 7 parents  6 therapists |  |
| 4. There was a prioritising of the objectives and interventions in collaboration with the child and his/her family. |  |  |  | 7 parents  7 therapists |  |
| 5. An effort was made to avoid overloading parents with information and exercises. |  | 2 therapists | 1 parent  3 therapists | 6 parents  2 therapists |  |
| 6. The therapist took the time needed with the family. |  |  | 1 therapist | 7 parents  6 therapists |  |
| 7. There was a focus on the positive. |  | 1 therapist | 1 parent  3 therapists | 6 parents  3 therapists |  |
| 8. There was teamwork. |  |  | 1 parent  5 therapists | 4 parents  2 therapists | 1 parent |
| 9. The family was provided with the support needed to engage in the services. |  | 4 therapists | 1 parent  2 therapists | 5 parents  1 therapist | 1 parent |
