# Supplementary material for: Evaluating a Family Capacity-Building Service: Are We Doing More Good Than Harm?
Source: Can J Occup Ther. 2025 Mar 13;92(2):113–25. doi: 10.1177/00084174251323729 (PMC12117127; doi:10.1177/00084174251323729)
Supplement: sj-docx-3-cjo-10.1177_00084174251323729 - Supplemental material for Evaluating a Family Capacity-Building Service: Are We Doing More Good Than Harm? [file sj-docx-3-cjo-10.1177_00084174251323729.docx]

Appendix C. Occupational therapists’ perspectives on the services in the MPOC-SP (Woodside et al., 2001)

|  | OT1 | OT2 | OT3 | Mean |
| --- | --- | --- | --- | --- |
| Showing interpersonal sensitivity | 5 | 5.2 | 4.8 | 5 |
| Providing general information | 1.4 | 1.4 | 1 | 1.27 |
| Communicating specific information | 6.67 | 6.33 | 3.33 | 4.44 |
| Treating people respectfully | 5.78 | 6.56 | 6.22 | 6.19 |

Note: For each category, seven represents the highest possible score. 1: not at all, 2: to a very small extent, 3: to a small extent, 4: to a moderate extent, 5: to a fairly great extent, 6: to a great extent, 7: to a very great extent.
