# Supplementary material for: Evaluating a Family Capacity-Building Service: Are We Doing More Good Than Harm?
Source: Can J Occup Ther. 2025 Mar 13;92(2):113–25. doi: 10.1177/00084174251323729 (PMC12117127; doi:10.1177/00084174251323729)
Supplement: sj-docx-4-cjo-10.1177_00084174251323729 - Supplemental material for Evaluating a Family Capacity-Building Service: Are We Doing More Good Than Harm? [file sj-docx-4-cjo-10.1177_00084174251323729.docx]

Appendix D. Parents’ perspectives on the services in the MPOC-20 (King et al., 2004)

|  | F1 | F2 | F3 | F4 | F5 | F6 | F7 | Mean |
| --- | --- | --- | --- | --- | --- | --- | --- | --- |
| Enabling and partnership | Invalid | Invalid | 6.67 | 7 | 7 | 7 | 7 | 6.93 |
| Providing general information | Invalid | 4 | Invalid | 3.6 | 6.8 | 5.6 | 7 | 5.4 |
| Providing specific information about the child | Invalid | Invalid | 7 | 6.67 | 6.67 | 7 | 7 | 6.87 |
| Coordinated and comprehensive care for the child and family | Invalid | 6.75 | 7 | Invalid | 6.75 | 5.25 | 7 | 6.55 |
| Respectful and supportive care | Invalid | 7 | 6.6 | 6.8 | 6.6 | 7 | 7 | 6.83 |

Note 1: For each category, seven represents the highest possible score. 1: never, 2: almost never, 3: from time to time, 4: sometimes, 5: to a fairly great extent, 6: to a great extent, 7: to a very great extent.

Note 2: When more than two-thirds of participants’ answers were 0 (“not applicable”), the subtest was considered invalid. The calculation of the mean does not include invalid subtests.

Note 3: The mixing of frequency and extent in the French version of the MPOC-20 is from the tool provided in French. While this issue appeared fixed in the English version according to King et al. (2004), it appears that the French version did not benefit from this improvement.
